# Predicting multiple sclerosis prognosis using AI and machine learning: integrating clinical, immunological, and radiological variables

**DOI:** 10.3389/fneur.2025.1712953

**Published:** 2026-01-15

**Authors:** Suhail Al-Shammri, Ahmet Özdil, Amro Aboukoura, Fawaz Azizieh, Bulent Yilmaz, Raj Raghupathy

**Affiliations:** 1 Department of Medicine, College of Medicine, Kuwait University, Safat, Kuwait; 2 Department of Computer Engineering, OSTİM Technical University, Ankara, Türkiye; 3 Division of Neurology, Mubarak Al-Kabeer Hospital, Ministry of Health, Jabriya, Kuwait; 4 College of Integrative Studies, Abdullah Al Salem University, Khaldiya, Kuwait; 5 Department of Electrical Engineering, Gulf University for Science and Technology, Hawally, Kuwait; 6 GUST Engineering and Applied Innovation Research (GEAR) Center, Gulf University for Science and Technology, Hawally, Kuwait; 7 Department of Microbiology, College of Medicine, Kuwait University, Safat, Kuwait

**Keywords:** EDSS, machine learning, MRI, multiple sclerosis, prediction, sensitivity, specificity

## Abstract

**Introduction:**

Accurate prediction of disease progression in multiple sclerosis (MS) remains a critical challenge in clinical management. This study investigates the utility of supervised machine learning (ML) models in predicting clinical disability, as measured by the Expanded Disability Status Scale (EDSS), and radiological activity based on MRI lesion changes in patients with relapsing-remitting MS (RRMS).

**Methods:**

Using peripheral cytokine profiles (IL-12, TNF-α, IFN-γ, IL-4, IL-10) along with patient metadata (e.g., sex, family history, relapse status), 43 ML classifiers were trained and evaluated for their ability to discriminate between mild and moderate disability (EDSS <1 vs >1, and <2.5 vs >2.5), and to predict new MRI lesions in 15 MS patients.

**Results:**

Ensemble models consistently outperformed simpler algorithms. For EDSS prediction, Random Forest achieved 90.1% sensitivity and 89.7% specificity, while Simple Logistic Regression reached 92.6% for both metrics when patient ID was included. In predicting new MRI lesions, Random Subspace classifiers performed best, with 82.4% sensitivity and specificity.

**Discussion:**

These findings suggest that combining cytokine-based immune signatures with machine learning strategies can provide clinically meaningful predictions of both functional disability and radiological progression. Such tools may support more proactive patient monitoring, informed therapeutic decision-making, and risk stratification in the care of RRMS. Further validation in prospective cohorts is warranted to support clinical implementation.

## 1 Introduction

Multiple sclerosis (MS) is a chronic, immune-mediated neuroinflammatory disorder of the central nervous system characterized by unpredictable disease trajectories and significant inter-individual variability in clinical outcomes (1). MS primarily affects individuals aged 20–40, with a higher prevalence in females (3:1 ratio) (2). The most common phenotype is relapsing–remitting MS (RRMS), which can evolve into secondary progressive MS (SPMS). Other forms include primary progressive MS (PPMS), clinically isolated syndrome (CIS), and radiologically isolated syndrome (RIS) (1, 3).

The transition from RRMS to SPMS and the variable rate of disability accumulation pose critical challenges to individualized patient management (4). Although current prognostic tools leverage clinical scales such as the Expanded Disability Status Scale (EDSS) and MRI-based lesion burden (5, 6), these measures often fail to fully capture the underlying pathophysiological processes driving disease progression, including subclinical inflammation, compartmentalized tissue injury, and neurodegeneration (7). RRMS is typically characterized by acute relapses followed by periods of remission, while progression independent of relapse activity (PIRA) becomes more prominent as patients advance to SPMS (8, 9). Disability is commonly monitored using the EDSS, although it does not adequately reflects cognitive, fatigue-related, and upper limb impairments (6). MRI remains pivotal in diagnosing and monitoring MS, with typical findings including T2- and FLAIR-hyperintense lesions, gadolinium-enhancing lesions (active inflammation), and T1 hypointensities (chronic damage) (10). Yet, the well-described clinico-radiological paradox underscores the weak correlation between lesion load and disability, particularly in later stages, prompting the need for additional biomarkers to capture disease heterogeneity (11).

The immunopathogenesis of MS involves autoreactive T cells crossing the blood–brain barrier, initiating inflammatory cascades that drive myelin and axonal damage. Pro-inflammatory cytokines such as interferon-gamma (IFN-γ), tumor necrosis factor-alpha (TNF-α), and interleukin-17 (IL-17) promote lesion development and neurodegeneration, while anti-inflammatory mediators like interleukin-10 (IL-10) and transforming growth factor-beta (TGF-β) tend to dampen the immune response (12). Imbalances between these pro- and anti-inflammatory signals are believed to underlie chronic, smoldering inflammation, particularly in progressive MS, contributing to disability accrual independent of overt relapse activity (12–15).

Recent advances in artificial intelligence (AI) and machine learning (ML) have shown significant promise in forecasting disease progression in multiple sclerosis (MS) by leveraging longitudinal clinical and imaging data (16–19). From an immunological perspective, Goyal et al. examined serum levels of eight cytokines, IL-1β, IL-2, IL-4, IL-8, IL-10, IL-13, IFN-γ, and TNF-α, in addition to demographic and clinical variables (13). Using various ML techniques, they reported that an ensemble random forest model achieved approximately 91% sensitivity in diagnosing MS and around 70% in distinguishing remitting from non-remitting disease, underscoring the prognostic utility of serum cytokine profiles. In a related study, patients with progressive MS exhibited elevated serum concentrations of pro-inflammatory cytokines (TNF-α, IFN-γ, IL-17) compared to both controls and individuals with relapsing–remitting MS (14). Notably, IL-10 was inversely correlated with disability, suggesting a potential protective role. These findings highlight a complex cytokine imbalance in progressive MS and reaffirm the involvement of inflammatory mediators in disease advancement. They further support the idea that peripheral cytokine profiles may serve as accessible biomarkers for subclinical central nervous system (CNS) inflammation. However, individual variability and the influence of multiple confounding factors limit the predictive accuracy of cytokines in isolation. This underscores the need for integrative, multimodal approaches that combine clinical, radiological, and immunological data to enhance predictive modeling (14, 19, 20).

Therefore, the primary aim of this study was to develop machine learning models capable of predicting both disability status (EDSS) and MRI lesion activity in MS patients by integrating a comprehensive dataset comprising: (1) cytokine profiles (comprising of three pro-inflammatory Th1 cytokines, IL-12, TNF-α, IFN-γ, and two anti-inflammatory Th2 cytokines: IL-4, IL-10). (2) gender, (3) family history of MS, (4) relapse rates, and (5) MRI lesion burden. By applying supervised learning algorithms to longitudinal data, we sought to determine whether peripheral cytokine signatures could serve as early predictive markers of both clinical disability (EDSS) and radiological activity (new or active lesions). Our study ultimately aims to inform the development of AI-driven, multimodal predictive tools that can enhance early risk stratification, guide personalized treatment strategies, and optimize therapeutic interventions in MS.

## 2 Methods

### 2.1 Subjects

The cohorts under investigation have not been previously described. In this observational study, at the turn of the millennium, we recruited 15 MS patients, all with a relapsing–remitting phenotype. The patients were diagnosed as per the Poser/McDonald criteria (21) and followed up by our team of experienced neurologists in the national MS clinic at Mubarak Al-Kabeer Hospital in Kuwait. They were seen over 4–5 points in time between the years 2000–2004, making a total number of 68 visits (time points). Patients were recruited into the study after providing voluntary informed consent (using a consent form approved by the Institutional Research Ethics Committee of the Health Sciences Centre, Kuwait University, Kuwait). At the time of recruitment, all subjects were on Interferon beta-1a (IFN-β-1a) as a disease-modifying therapy (DMT). Information collected were on demographic parameters, including age, gender, and ethnicity. Moreover, clinical parameters such as disease duration, family history of MS, number of relapses, disease severity, and MRI lesion load were recorded at baseline. Indicators of disease activity and progression — including the occurrence of new relapses, new or active MRI lesions, and changes in EDSS — were monitored during each scheduled or unscheduled outpatient visit over a follow-up period of 2 to 3 years. Disabilities were stratified according to the expanded disability status scale (EDSS) (3). The EDSS rates the clinical status of a patient on a 0–10 scale, where a score of 0 indicates that the patient is normal, whereas a score of 10 denotes death. A relapse was defined as the emergence of new or previous signs or symptoms that lasted for more than 24 h (3). In terms of MRI features, active MS disease is considered if there is appearance of new lesions or detection of enhancing lesions after gadolinium infusion, both in the brain and spinal cord.

Given that the majority of EDSS values in this cohort fell within the low-disability range (0–2.5) and only two patients reached scores above 3.0 (EDSS 4.0 and 4.5 during follow-up), EDSS was dichotomized in two ways for subsequent analyses: <1 vs. >1 and <2.5 vs. >2.5. EDSS >1 was used to capture the earliest transition from normal or minimally symptomatic status to measurable neurological impairment, whereas EDSS >2.5 was used to mark the emergence of more widespread, multi-system deficits, preceding the classical EDSS ≥3.0 disability milestone. These dichotomies thus preserved clinical relevance while maintaining sufficient event numbers for robust model training in this small longitudinal dataset.

MRI examinations were performed as part of routine clinical care using 1.5 T scanners. Standard MS protocols were applied, including FLAIR, T2-weighted, and post-contrast T1-weighted sequences when gadolinium was clinically indicated. Lesion scoring was performed by experienced MS radiologists who were blinded to cytokine and other clinical data. Active disease on MRI was defined according to the MRI components of the McDonald criteria for dissemination in space (DIS) and dissemination in time (DIT), requiring new, enlarging, or gadolinium-enhancing lesions in the brain or spinal cord, with confirmation on follow-up scans when available. For the machine learning analyses, MRI activity at each visit was coded as a binary outcome (active vs. non-active) based on these criteria.

### 2.2 Mitogen-induced stimulation of peripheral blood mononuclear cells (PBMC)

Blood was obtained via venipuncture, and peripheral blood mononuclear cells (PBMC) were isolated using Ficoll-paque density gradient centrifugation (Pharmacia Biotech, Sweden). The isolated PBMCs were suspended in RPMI medium (GIBCOBRL, USA) supplemented with 10% fetal calf serum. The cells were then aliquoted into 96-well tissue culture plates at a density of 10^5^ cells per well and stimulated with phytohemagglutinin (PHA) (Sigma Chemicals, USA) at a concentration of 5 μg/mL for 96 h. Supernatants were collected on day 4 and were stored at −80 °C for subsequent cytokine estimation.

### 2.3 ELISA for estimation of cytokine levels

Quantification of cytokine levels was conducted using sandwich enzyme-linked immunosorbent assay (ELISA) kits obtained from Immunotech SA (France). The manufacturer’s protocols were followed for the assays. Duplicate samples were tested, and absorbance values were measured using an ELISA Reader. Accurate concentrations of cytokines in the samples were determined by comparing their absorbance values with those obtained for the reference standards plotted on a standard curve, utilizing reference recombinant cytokines. The cytokine profile included the Th1 cytokines IL-12, TNF-α, and IFN-γ and the Th2 cytokines IL-4 and, IL-10. All standards, controls, and samples were measured in duplicates. The sensitivity of each assay was as follows: 5 pg./mL for IL-2, 10 pg./mL for TNF-α, 3 pg./mL for IFN-γ, 5 pg./mL for IL-4, and 5 pg./mL for IL-10. In accordance with standard procedures, any cytokine concentration values that were recorded as zero or deemed undetectable were replaced with the minimum detectable value (sensitivity).

### 2.4 Statistical analysis

Statistical analysis was conducted using the Statistics and Machine Learning Toolbox in MATLAB (version 2023b). Initially, Shapiro–Wilk normality test was applied to the cytokine concentration values in each group, revealing that none of the distributions were normal. Therefore, a nonparametric test, the Wilcoxon rank-sum test, was used.

### 2.5 Dataset preparation for machine learning of EDSS score

Table 1 presents data from 68 cases, with 32 (47%) having an EDSS of 0 or 1 (≤1) and 36 (53%) having a score higher than 1 (>1). Each case corresponded to a set of five cytokine values recorded during a single visit, along with all other related clinical information. The table also details the number of cases for each specific EDSS score. Notably, most of the cases (58/68; 85%) had EDSS scores of 0, 1, 1.5, or 2 (<2.5); while the remaining (10/98; 15%) had EDSS ≥2.5.

**Table 1:** Distribution of samples by EDSS (expanded disability status scale) score.

EDSS score	Number of samples
0	15
1	17
1.5	14
2	12
2.5	4
3.5	2
6	4

For the binary classification phase of this study, which focuses on EDSS scores using cytokine profiles, scores of ≤1 were assigned to the first category, while scores >1 were assigned to the second category. This is referred to as “test setup 1.” A similar classification task used EDSS scores of <2.5 as the first category and ≥2.5 values as the second category, which is referred to as “test setup 2.”

In addition to the 5 cytokine concentration values, gender, the existence of family history and relapse, and observation of new lesions on the MRIs were included as numerical features (1 for existent, 0 for nonexistent) for classifying EDSS scores using various machine learning approaches. These 9 values comprised the 9-dimensional feature space for this classification task (attributes).

### 2.6 Dataset preparation for machine learning of MRI findings

There were 43 (63%) cases labeled as either ‘no lesion’ or ‘stable,’ and 25 (37%) cases with at least one new lesion (up to 4 new lesions) determined by examining the patients’ MR images. For the binary classification phase of this study, which focused on detecting MRI, labels of ‘no lesion’ or ‘stable’ were assigned to the first category (STABLE), while labels of ‘one new lesion’ or ‘multiple new lesions’ were assigned to the second category (NEW LESION). In addition to the 5 cytokine concentration values, gender of the patient, the existence of family history and relapse (1 for existent, 0 for nonexistent), as well as whether the EDSS score was ≤1 (label 0) vs. >1 (label 1), were included as the features/attributes for classifying MRI labels using various machine learning approaches (total 9 attributes). This is referred to as “test setup 3″. We used the similar classification task of EDSS score <2.5 (label 0) vs. ≥2.5 (label 1), the test is referred to as “test setup 4″ and applied the same 9-dimensional feature space for this classification task. Table 2 summarizes the binary classification and attributes for each of the test setups.

**Table 2:** Binary classification and attributes for each test setup.

Test setup	Binary classification	Attributes
Test setup 1	EDSS ≤ 1 vs. EDSS > 1	5 cytokine concentration values, Gender, Family history, Relapse, MRI finding (STABLE vs. NEW LESION)
Test setup 2	EDSS < 2.5 vs. EDSS ≥ 2.5	
Test setup 3	MRI STABLE vs. NEW LESION	5 cytokine concentration values, Gender, Family history, Relapse, EDSS (≤1 vs. EDSS>1)
Test setup 4	MRI STABLE vs. NEW LESION	5 cytokine concentration values, Gender, Family history, Relapse, EDSS (<2.5 vs. EDSS ≥ 2.5)

### 2.7 Machine learning techniques

In this study, the open-source WEKA (Waikato Environment for Knowledge Analysis - University of Waikato in New Zealand) software package, which offers several visualization tools and algorithms for data analysis and predictive modeling, is used for machine learning (ML) methods evaluations (22). We conducted four different binary classification tasks as explained above (Table 2). The cytokine concentration values and other relevant information such as the gender, existence of family history and relapse, and new lesion detected on MR images or EDSS scores were used as the attributes/features for these classification tasks.

We investigated the performance and feasibility of 43 machine learning techniques such as Naïve Bayes Multinomial Text, Cross Validation Parameter Selection, Multi Scheme, Stacking, Voting, etc. The performance of all 43 methods was analyzed and the ones which stand out among other machine learning approaches available in WEKA were reported in the Results section. The best performing methods that are listed above are probabilistic and/or ensemble approaches. To evaluate the performance of the machine learning models and prevent any potential data leakage, we employed a leave-one-patient-out (LOPO) approach. In this method, the data from one patient served as the validation set while the data from all remaining patients were used for training. This process was repeated iteratively for each patient in the dataset.

### 2.8 Performance metrics

We evaluated the performance of our classification models using micro-averaged Average Precision (AP), and micro-averaged Area Under the Precision-Recall Curve (AUPRC) computed with trapezoidal integration. Micro-averaged Average Precision (micro-AP) was employed to evaluate the overall ranking performance of the models. Unlike macro-averaging, which computes the Average Precision (AP) independently for each class and then averages the results, micro-AP aggregates all predictions across all classes and treats them as a single pool of binary decisions. This approach accounts for every instance–label pair equally, thereby weighting classes proportionally to their frequency in the dataset. Micro-AP is calculated by sorting all predicted confidence scores in descending order and computing the area under the resulting precision–recall curve, where precision is evaluated at every rank position where a true positive occurs. As a result, micro-AP provides a robust and informative measure of a model’s ability to globally rank true positive instances ahead of false positives, particularly in datasets with substantial class imbalance. Instead of threshold-based metrics, we report micro-averaged AP with bootstrap confidence intervals, which remains stable under leave-one-patient-out regardless of class imbalance per patient. Micro-AUPRC is computed by applying the trapezoidal rule to the precision–recall curve and produces the area under the interpolated curve that connects precision–recall points with straight lines. Micro-AP differs from micro-AUPRC computed with the trapezoidal rule in that micro-AP uses a stepwise interpolation based on precision at each true-positive rank, whereas trapezoidal AUPRC linearly interpolates between points, typically yielding a lower area. These metrics ensure a robust evaluation of the performance of our models by capturing various aspects of predictive accuracy.

## 3 Results

The demographic and baseline clinical characteristics of the subjects enrolled in the study are summarized in Table 3. Data are presented as mean ± standard deviation (SD) to reflect the central tendency and variability within the sample population. These baseline characteristics provide essential context for interpreting the study outcomes and understanding the clinical profile of the participants. There were no statistical differences in any of the demographics and clinical characteristics between males and females in this study.

**Table 3:** Demographics and baseline clinical characteristics of subjects enrolled in the study (mean ± SD).

Characteristic	Total (*N* = 15)	Female (*N* = 9)	Male (*N* = 6)	*p*-value
Age, years (mean ± SD)	33.0 ± 9.5	33.2 ± 10.1	31.0 ± 9.5	0.84 (ns)
Ethnicity, *N* (%)				
Arabs	13 (86.7%)	7 (77.8%)	6 (100%)	0.49 (ns)
Persians	2 (13.3%)	2 (22.2%)	0	—
Family history of MS, *N* (%)				
Negative	10 (66.7%)	7 (77.8%)	3 (50%)	0.33 (ns)
Positive	5 (33.3%)	2 (22.2%)	3 (50%)	
MS type, *N* (%)				
RRMS	15 (100%)	9 (100%)	6 (100%)	—
SPMS (current or last follow-up)	7 (46.7%)	5 (55.6%)	2 (33.3%)	0.61 (ns)
MS duration, years (mean ± SD)	25.3 ± 7.8	26.1 ± 8.0	24.1 ± 7.5	0.52 (ns)
Number of relapses at baseline, median (IQR)	3 [2–5]	3 [2–5]	4 [2–5]	0.68 (ns)
Total relapses, median (IQR)	6 [5–7]	6 [5–7]	7 [6–7]	0.72 (ns)
EDSS at baseline, median (IQR)	1.5 [1–2]	1.5 [1–2]	1.5 [1–2]	0.90 (ns)
EDSS at last follow-up, median (IQR)	4 [1–6]	4 [1–6]	4 [1–6]	0.95 (ns)

### 3.1 Comparison of individual cytokine levels between study groups

**Figure 1:**
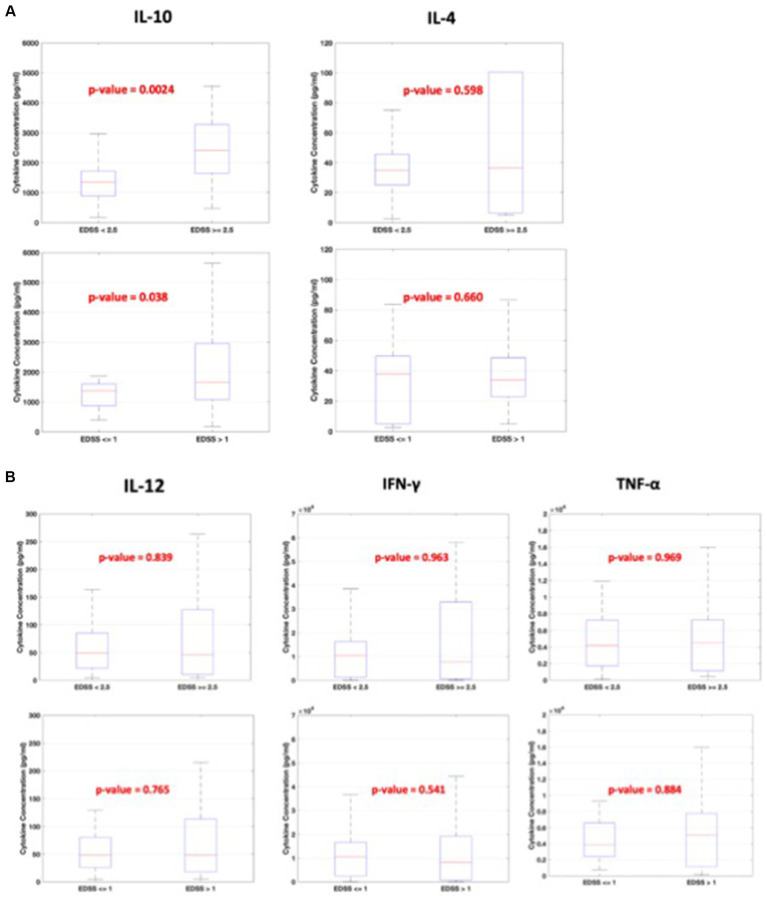
**(A,B)** The descriptive statistics for the concentrations of all cytokines calculated separately for patients EDSS scores <2.5 vs. ≥2.5 and EDSS scores ≤1 vs. >1.

**Figure 2:**
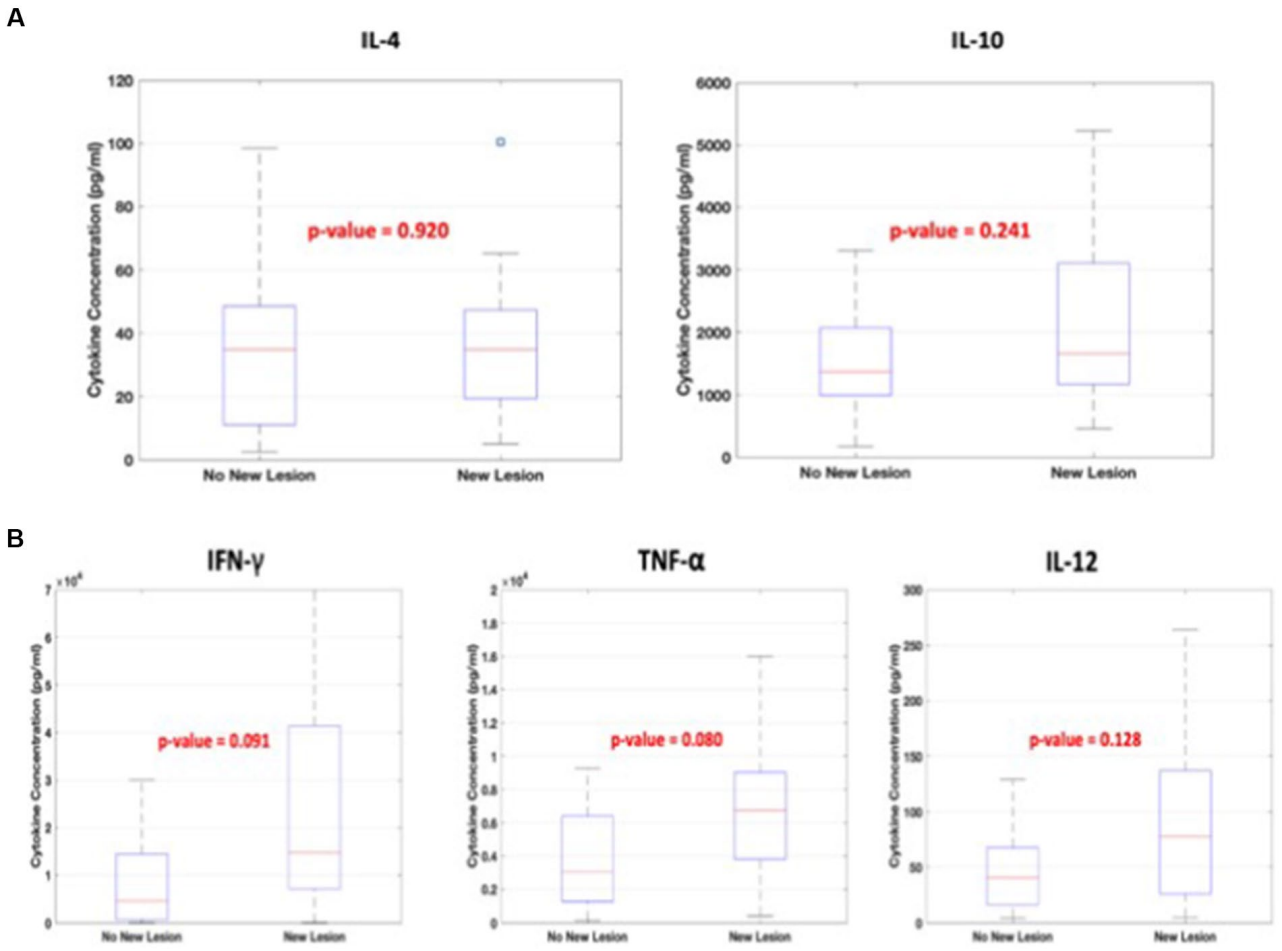
**(A,B)** The descriptive statistics for the concentrations of all cytokines calculated separately for patients with and without new lesions on MRI.

The statistical comparison of concentration values of each cytokine type between the groups with EDSS scores ≤1 vs. >1 and EDSS scores <2.5 vs. ≥2.5 was performed. In addition, a similar comparison was performed between the cases with NEW LESION or STABLE on MRI. Among the pro- and anti-inflammatory cytokines measured, only IL-10 showed a statistically significant difference (*p* < 0.05) between high and low EDSS scores in both cases (EDSS scores ≤1 vs. >1 and EDSS scores <2.5 vs. ≥2.5) as shown in Figures 1A,B. On the other hand, the differences in cytokine concentration values between (NEW LESION) and (STABLE) groups showed no statistical significance (*p* > 0.05) as shown in Figures 2A,B.

### 3.2 Machine learning based classification results for EDSS

**Figure 3:**
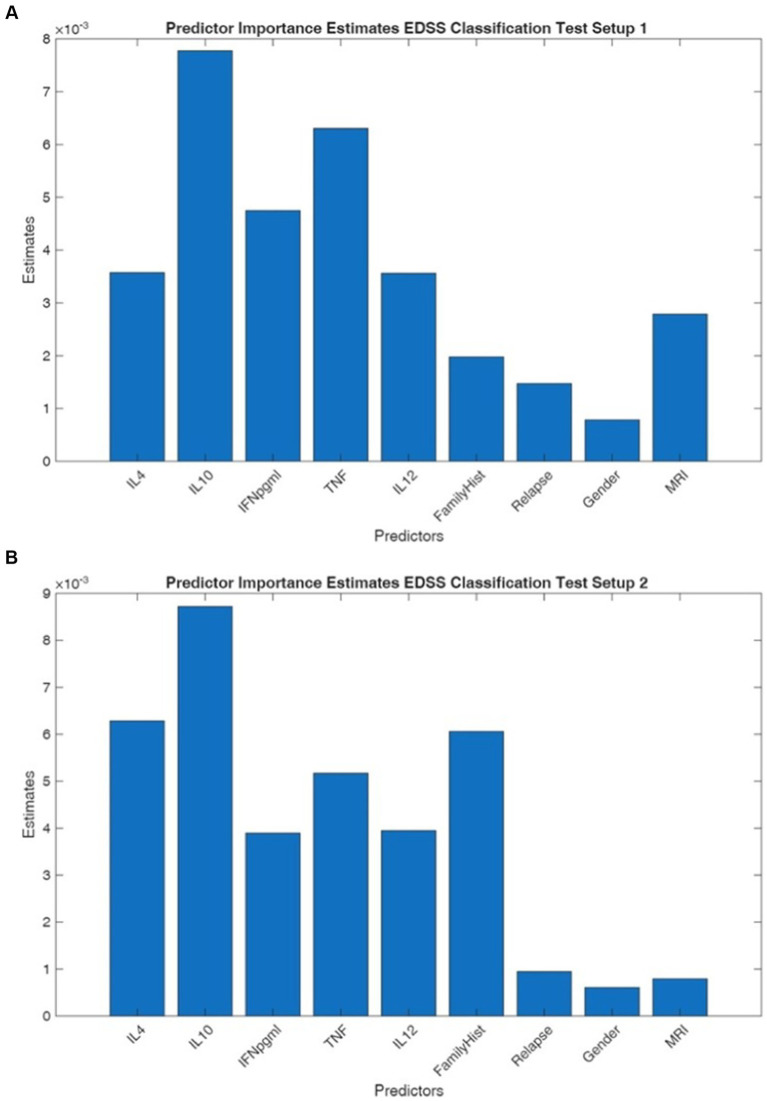
**(A,B)** The predictor importance estimates for the discrimination of patients with EDSS scores ≤1 vs. >1 (test setup 1, left panel) and EDSS scores (<2.5 vs. >2.5; test setup 2, right panel). In EDSS classification (test setup 1 and 2), IL-10 consistently emerged as the most important predictor with estimates of 0.0078 and 0.0087, respectively, followed by TNF-α (0.0063 and 0.0051) and IL-4 (0.0036 and 0.0063). Family history and IFN-γ showed moderate importance (ranging from 0.0039 to 0.0060), while IL-12 demonstrated variable importance between setups (0.0035 to 0.0040).

The results of the binary classification tasks focusing on EDSS score prediction are presented in Table 4, showcasing the performance metrics for each methodology employed. We conducted a binary classification task to determine whether the Expanded Disability Status Scale (EDSS) score is > 1 (test setup 1) or ≥ 2.5 (test setup 2) can be predicted, using cytokine concentration values and other relevant information such as the gender, existence of family history and relapse, and new lesion detected on MR images. Additionally, we assessed the performance of 43 machine learning techniques and found that the Naïve Bayes Multinomial Text, Cross Validation Parameter Selection, Multi Scheme, Stacking and Voting models were the best performing models, based on micro-averaged Average Precision (micro-AP). With AP values between 0.97–0.98 in test setup 1 (EDSS <1 vs. >1) and between 0.93–0.94 in test setup 2 (EDSS <2.5 vs. >2.5), these models exhibit extremely good ranking performance for both classification tasks regarding EDSS prediction. Their overlapping 95% confidence intervals and small fluctuation in AUPRC (computed with the trapezoidal rule) show that they continuously favor positive samples at the top of the ranking and act similarly. This implies that the underlying feature distributions are sufficiently discriminative for these probabilistic and ensemble approaches to effectively utilize the cytokine-based classification tasks represented by test setups 1 and 2. These tests show great separability and dependable model performance, as seen by the high AP values and confidence limits considerably over 0.90 (Figures 3A,B).

**Table 4:** Results of the binary classification tasks for EDSS score.

Test setup	ML Technique	AP	AUPRC	AP CI (2.5%)	AP CI (97.5%)
Test setup 1: EDSS (≤1 vs. >1)	Naïve Bayes	0.9766	0.9764	0.9153	0.9969
Test setup 1: EDSS (≤1 vs. >1)	CV Parameter Selection	0.9766	0.9764	0.9200	0.9973
Test setup 1: EDSS (≤1 vs. >1)	Multi Scheme	0.9766	0.9764	0.9066	0.9970
Test setup 1: EDSS (≤1 vs. >1)	Stacking	0.9766	0.9764	0.9073	0.9966
Test setup 1: EDSS (≤1 vs. >1)	Voting	0.9766	0.9764	0.9098	0.9973
Test setup 2: EDSS (<2.5 vs. ≥2.5)	Naïve Bayes	0.9379	0.9366	0.5751	0.9935
Test setup 2: EDSS (<2.5 vs. ≥2.5)	CV Parameter Selection	0.9379	0.9366	0.5546	0.9939
Test setup 2: EDSS (<2.5 vs. ≥2.5)	Multi Scheme	0.9379	0.9366	0.5556	0.9936
Test setup 2: EDSS (<2.5 vs. ≥2.5)	Stacking	0.9379	0.9366	0.5409	0.9945
Test setup 2: EDSS (<2.5 vs. ≥2.5)	Voting	0.9379	0.9366	0.5556	0.9918

### 3.3 Machine learning based classification results for MRI findings

In this part of the study, we conducted a binary classification task to determine whether MRI results can be predicted (STABLE vs. NEW LESION) using the same dataset prepared by thresholding the EDSS score from >1 and ≥2.5 levels for test setup 3 and 4, respectively (Table 2), using cytokine concentration values and other relevant information such as the gender, existence of family history and relapse, and EDSS scores. Additionally, we assessed the performance of 43 machine learning techniques and found that the Naïve Bayes Multinomial Text, Cross Validation Parameter Selection, Multi Scheme, Stacking and Voting models were the best performing models, based on micro-averaged Average Precision (micro-AP).

**Figure 4:**
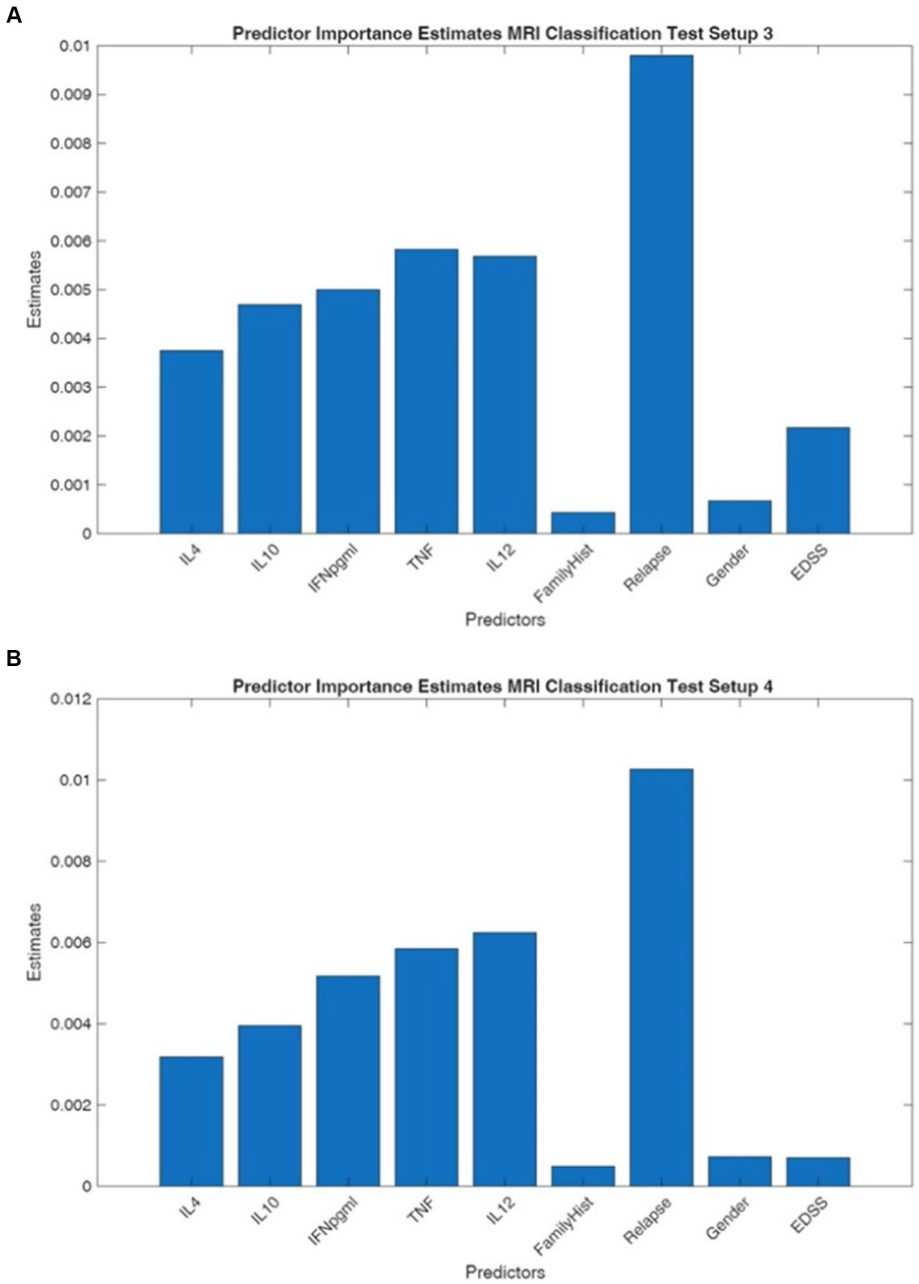
**(A,B)** The predictor importance estimates for the discrimination of MRI results as stable vs. new lesion patients with EDSS scores ≤1 vs. >1 (test setup 3, left panel) and EDSS scores (<2.5 vs. >2.5; test setup 4, right panel).

In Table 5, the ability of each classifier to discriminate between classes is demonstrated by the by the micro-AP and micro-AUPRC values which did not vary significantly across models and cases. In test setups 3 and 4, where the goal was to predict the existence of MRI lesions using EDSS ≤1 vs. >1 and EDSS <2.5 vs. >2.5 respectively, show significantly lower AP values with larger and more varied confidence ranges, both centered around 0.62 (Figures 4A,B). This decline suggests a more difficult categorization scenario with perhaps more patient-level variability and a less identifiable signal. Despite this challenge, the relative ranking of the top models is consistent: the top model families continue to perform at the highest levels, but the variation between them is significantly smaller.

**Table 5:** Results of the binary classification tasks lesion detection in MRI.

Test setup	ML technique	AP	AUPRC	AP CI (2.5%)	AP CI (97.5%)
Test setup 3: Lesion detection in MRI EDSS (≤1 vs. >1)	Naïve Bayes	0.6238	0.6102	0.4726	0.6964
Test setup 3: Lesion detection in MRI EDSS (≤1 vs. >1)	CV Parameter Selection	0.6238	0.6102	0.4779	0.6964
Test setup 3: Lesion detection in MRI EDSS (≤1 vs. >1)	Multi Scheme	0.6238	0.6102	0.4795	0.6958
Test setup 3: Lesion detection in MRI EDSS (≤1 vs. >1)	Stacking	0.6238	0.6102	0.4748	0.6942
Test setup 3: Lesion detection in MRI EDSS (≤1 vs. >1)	Voting	0.6238	0.6102	0.4756	0.6940
Test setup 4: Lesion detection in MRI EDSS (<2.5 vs. ≥2.5)	Naïve Bayes	0.6238	0.6102	0.4783	0.6961
Test setup 4: Lesion detection in MRI EDSS (<2.5 vs. ≥2.5)	CV Parameter Selection	0.6238	0.6102	0.4755	0.7023
Test setup 4: Lesion detection in MRI EDSS (<2.5 vs. ≥2.5)	Multi Scheme	0.6238	0.6102	0.4747	0.6989
Test setup 4: Lesion detection in MRI EDSS (<2.5 vs. ≥2.5)	Stacking	0.6238	0.6102	0.4735	0.7004
Test setup 2: Lesion detection in MRI EDSS (<2.5 vs. ≥2.5)	Voting	0.6238	0.6102	0.4800	0.6993

MRI classification models (Test setups 3 and 4) identified Relapse as the dominant predictor with substantially higher importance estimates of 0.0098 and 0.0102, markedly exceeding all other predictors. Inflammatory cytokines maintained moderate importance in MRI classification, with TNF-α (0.0058 and 0.0058), IL-12 (0.0056 and 0.0062), and IFN-γ (0.0050 and 0.0052) showing consistent contributions. Demographic variables including Gender, Family History, and EDSS consistently showed minimal predictive value across all test setups, with estimates typically below 0.0025.

## 4 Discussion

This study evaluated the utility of machine learning (ML) models in predicting disease progression in multiple sclerosis (MS) by integrating MRI parameters, clinical data, and cytokine profiles. Our findings highlight the promising role of ML-based predictive frameworks in enhancing the prognostic sensitivity for MS, especially when traditional clinical metrics such as EDSS, relapse rate, and lesion load are complemented by immunological biomarkers.

A few recent reports have described serum cytokine levels in relation to MS (12–15). While these findings are informative, it is important to recognize that serum cytokine concentrations represent only a small fraction of the overall cytokine produced by peripheral leukocytes or relevant tissues. Additionally, various patient-related factors such as age, gender, circadian rhythm, food intake, physical activity, and stress—can also contribute to variability in serum cytokine measurements (23). Given these limitations, systemic inflammatory status is more accurately assessed by profiling cytokines produced by mitogen-stimulated peripheral blood mononuclear cells (PBMCs) ex vivo (24, 25). This method offers a more functional assessment of immune responsiveness, reflecting the capacity of PBMCs to produce cytokines in response to stimulation.

Importantly, our study found that IL-10—an anti-inflammatory cytokine—was significantly elevated in patients with higher EDSS scores when dichotomized as ≤1 versus >1 and <2.5 versus ≥2.5. This is a compelling, albeit counterintuitive, finding, as one might expect anti-inflammatory cytokines like IL-10 to be associated with lower disease burden. However, elevated IL-10 levels may reflect a compensatory response to ongoing inflammatory activity or a shift toward immune dysregulation and exhaustion in patients with more advanced disability. It is also possible that peripheral IL-10 does not directly mirror CNS inflammation, highlighting the complex dynamics of immune regulation in MS.

While our finding of elevated IL-10 in patients with higher EDSS may reflect a compensatory immunoregulatory response or immune exhaustion, alternative explanations merit careful consideration. Firstly, all individuals in our cohort were treated with interferon-β, which has been shown to modulate IL-10 expression. The mechanisms of action of IFN-β in MS include effects on IL-10 production, potentially contributing to higher systemic IL-10 independent of central nervous system (CNS) disease burden (26). Second, the IL-10/IL-10R axis is dynamically regulated by environmental and peripheral immune factors; increased peripheral IL-10 may be driven by systemic immune activation rather than CNS inflammation *per se* (27). Third, age-related immunosenescence may play a role. Aging in MS has been associated with elevated levels of IL-10, possibly as part of a broader “inflamm-aging” or immune remodeling (28). Thus, elevated production of IL-10 in patients with higher disability may not solely indicate compensatory immunoregulation but could also result from therapy, systemic immune shifts, or age-dependent immunological changes. Future work should involve the stratification of patients based on the treatment exposure, age and systemic inflammatory markers to dissect the relative contributions of these mechanisms.

Given that cytokines operate within an intricate and interdependent network, it is increasingly recognized that the level of a single cytokine provides limited insight into the overall immune state (29). This has prompted a shift in cytokine research from the analysis of individual cytokines or simple ratios to multivariate, many-cytokine signature approaches. Notable examples include studies by Azizieh et al., who investigated a panel of 10 cytokines in relation to bone mineral density in postmenopausal women (24); Heard et al., who profiled 38 cytokines in rheumatoid arthritis (30); Boute et al., who analyzed 56 intracellular cytokines for cancer immunotherapy monitoring (31); and Wei et al., who utilized a 93-cytokine profile in cancer immunomodulation (32). The next analytical frontier involves integrating immunological, clinical, and radiological data into multimodal models to better understand disease progression and response to treatment (13, 19, 20).

In parallel, systematic work applying machine learning to clinical EEG data in MS has emphasized both the promise and the methodological challenges of such approaches, underscoring the need for interpretable, multimodal models and rigorous external validation (33).

The application of AI to cytokine analysis holds immense promise for revolutionizing disease prediction, early diagnosis, and the development of personalized interventions. By leveraging the dynamic and sensitive nature of cytokine signatures, AI can uncover hidden patterns, predict disease outcomes, and guide tailored treatments. In general, artificial intelligence (AI), particularly machine learning and deep learning approaches, are extensively used in analyzing complex biomarker data. These techniques aim to detect hidden patterns, make predictions about disease progression, and assist in diagnosis and treatment. It is important to note that the methods used in biomarker analysis are also widely applied in other fields such as finance and technology (34).

This study evaluated the performance of different classification algorithms across four test setups, which may accommodate for the substantial inter-individual variability and consider patient-specific pattern based on individual trend rather than the general trend of all patients. The findings highlight that Naïve Bayes Multinomial Text, Cross-Validation Parameter Selection, Multi Scheme, Stacking, and Voting methods consistently outperformed others in terms of the performance criterion referred to as micro-averaged Average Precision (AP). making them the most reliable classifiers overall. The top-performing models belong to the same family of ensemble-based and probabilistic classifiers.

The strong and consistent performance of the ensemble-based and probabilistic models in test setups 1 and 2 highlights the presence of highly discriminative cytokine patterns within these test conditions. Their exceptionally high micro-averaged AP values—paired with tight confidence intervals and minimal variation in AUPRC—indicate that these methods can robustly identify and rank true-positive instances even under cross-validated LOPO evaluation, which is known to impose stricter generalization requirements. This pattern suggests that the underlying feature distributions in test setups 1 and 2 provide clear separation between classes, allowing probabilistic estimators and ensemble strategies to capitalize on subtle but stable shifts in cytokine levels. In contrast, the markedly lower AP values observed for test setups 3 and 4 point to more complex or heterogeneous biological dynamics, where the cytokine signatures are either weaker, noisier, or more patient-specific. Despite these more challenging conditions, the same model families remain the top performers, underscoring their ability to maintain relative stability even when the signal-to-noise ratio deteriorates. The grouping of results into two distinct performance regimes therefore reflects differences in biological separability across test configurations: one in which cytokine alterations are strong enough to support near-perfect discrimination, and another in which the biomarkers carry only partial predictive value. Collectively, these findings indicate both the strength of the modeling approaches and the variable informativeness of the cytokine profiles across different physiological or experimental contexts.

In this study, learning curves were not generated because the leave-one-patient-out (LOPO) procedure fixes the training set size for each fold, leaving no mechanism to vary the amount of training data. Instead, we quantified fold-wise performance variability using pooled bootstrap confidence intervals across patients. In addition, sensitivity and specificity could not be reliably estimated, as several LOPO test folds contain only positive or only negative samples for certain patients. In these folds, sensitivity or specificity is either undefined or exhibits zero variance. Consequently, rather than using threshold-dependent metrics, we report micro-averaged area under the precision-recall curve (AUPRC, or AP) with bootstrap confidence intervals, which provide a stable performance measure under LOPO irrespective of class imbalance at the patient level.

The divergent predictor importance patterns between EDSS and MRI classification models suggest that these outcome measures capture distinct aspects of disease pathophysiology. The prominence of IL-10 in EDSS prediction aligns with its established role as an anti-inflammatory cytokine that may modulate clinical disability progression, potentially reflecting systemic immune regulation mechanisms that influence functional outcomes. Conversely, the overwhelming importance of Relapse history in MRI classification indicates that radiological disease activity is more strongly associated with historical acute inflammatory events than with current cytokine profiles or baseline disability measures. This finding supports the concept that MRI lesion burden accumulates through recurrent inflammatory episodes, whereas disability progression may follow a more complex trajectory influenced by both inflammatory and neurodegenerative processes. The consistent moderate contribution of pro-inflammatory cytokines (TNF-α, IFN-γ) across both classification types suggests these markers capture disease activity relevant to both clinical and radiological outcomes, though their relative importance varies by endpoint. The minimal contribution of demographic variables across all models indicates that biological markers and clinical history provide substantially greater predictive value than age, gender, or family history alone, emphasizing the primacy of disease-specific factors in outcome prediction.

Our results align with recent studies that support the role of AI and ML in refining MS prognosis. Eshaghi et al. (16) used unsupervised machine learning to derive MRI-based subtypes of MS predictive of future disability accumulation, offering a data-driven complement to conventional clinical phenotyping. Similarly, La Rosa et al. (35) demonstrated that deep learning models trained on MRI could predict CIS conversion with high sensitivity, outperforming human readers. These studies, like ours, underscore the potential of AI to identify subtle patterns in imaging and immunological data that may escape clinical detection.

An important strength of our study is the incorporation of biological markers, specifically pro-inflammatory and anti-inflammatory cytokines, and the novel use of a five-cytokine signature as a predictive feature in AI/ML. This approach reflects the immunopathological heterogeneity of MS and recognizes the contribution of systemic inflammation to disease activity and progression (36). Few ML studies have incorporated longitudinal cytokine data into predictive models (13, 14, 16); thus, our study adds an important dimension to MS prognostication by bridging clinical, radiological, and immunological domains.

Moreover, an extended follow-up duration of up to 2–3 years enhances the robustness of our findings, providing insights into the longitudinal trajectory of MS in a real-world cohort. This extended observation period allowed for accurate characterization of disease progression, relapse dynamics, and treatment effects over time — factors that are often underrepresented in shorter studies. Our detailed AI/ML analysis incorporated not only baseline but also visit-level data, enabling the prediction of dynamic outcomes such as relapse risk and active MRI lesions, which strengthens the practical relevance of our findings.

Despite these strengths, our study has limitations that warrant consideration. The small sample size limits the generalizability of our results and may introduce overfitting in ML models. While we used feature selection, cross-validation, and model tuning strategies to mitigate this risk, external validation in larger, multicenter datasets is necessary to confirm model performance and reliability. Additionally, variability in MRI acquisition protocols over the follow-up period may affect the consistency of imaging features used in training the models. Future studies should standardize imaging parameters or apply domain adaptation techniques to account for such heterogeneity.

Given the retrospective nature of this study causal relationships between cytokine profiles and disease progression cannot be inferred directly. Our analysis is limited to identifying associations and generating predictive models based on existing clinical, immunological, and radiological variables, and a prospective longitudinal validation is required to confirm predictive performance of the model over time and to further investigate potential causal mechanisms.

In this study we measured the levels of only five cytokines. Future studies can be made more robust and unequivocal by including a larger panel of pro- and anti-inflammatory and regulatory cytokines as well as genetic, proteomic and clinical markers.

In addition, the models were evaluated on pre-defined test setups; however, external validation on independent datasets or prospective data is essential to confirm the models’ real-world applicability. Additionally, feature selection and preprocessing decisions (e.g., thresholding EDSS scores) may impact the reproducibility and interpretation of results. Lastly, the clinical interpretability of machine learning outcomes remains a challenge, particularly for ensemble methods, which may hinder adoption by healthcare practitioners unless complemented by explainable techniques.

## 5 Conclusion

In conclusion, our study provides compelling evidence for the potential of machine learning as a valuable adjunct to conventional prognostic tools in MS. By integrating clinical, radiological, and immunological data, with a particular emphasis on cytokine expression patterns and their predictive roles in relapse and MRI activity, our approach offers a more comprehensive framework for predicting disease progression. Future research endeavors should prioritize the validation of these models in larger, more diverse cohorts, focus on enhancing their interpretability, and explore the integration of additional biological markers, such as genetic or proteomic profiles, to further personalize the management of MS.

## Data Availability

The datasets presented in this study can be found in online repositories. The names of the repository/repositories and accession number(s) can be found at: https://figshare.com/account/items/29828933/edit.

## References

[1] Reich DS Lucchinetti CF Calabresi PA. Multiple Sclerosis. N Engl J Med. (2018) 378:169–80. doi: 10.1056/NEJMra1401483, 29320652 PMC6942519

[2] Portaccio E Magyari M Havrdova EK Ruet A Brochet B Scalfari A . Multiple sclerosis: emerging epidemiological trends and redefining the clinical course. Lancet Reg Health Europe. (2024) 44:977. doi: 10.1016/j.lanepe.2024.100977, 39444703 PMC11496978

[3] Vollmer TL Nair KV Williams IM Alvarez E. Multiple sclerosis phenotypes as a continuum: the role of neurologic reserve. Neurol Clin Pract. (2021) 11:342–51. doi: 10.1212/CPJ.0000000000001045, 34476126 PMC8382415

[4] Kleiter I Ayzenberg I Havla J Lukas C Penner I-K Stadelmann C . The transitional phase of multiple sclerosis: characterization and conceptual framework. Mult Scler Relat Disord. (2020) 44:102242. doi: 10.1016/j.msard.2020.102242, 32535501

[5] Kaunzner UW Gauthier SA. MRI in the assessment and monitoring of multiple sclerosis: an update on best practice. Ther Adv Neurol Disord. (2017) 10:247–61. doi: 10.1177/1756285617708911, 28607577 PMC5453402

[6] Kurtzke JF. Rating neurologic impairment in multiple sclerosis. Neurology. (1983) 33:1444–4. doi: 10.1212/WNL.33.11.1444, 6685237

[7] Koch MW Mostert J Repovic P Bowen JD Strijbis E Uitdehaag B . MRI brain volume loss, lesion burden, and clinical outcome in secondary progressive multiple sclerosis. Mult Scler. (2022) 28:561–72. doi: 10.1177/13524585211031801, 34304609 PMC8961253

[8] Kappos L Wolinsky JS Giovannoni G Arnold DL Wang Q Bernasconi C . Contribution of relapse-independent progression vs relapse-associated worsening to overall confirmed disability accumulation in typical relapsing multiple sclerosis in a pooled analysis of 2 randomized clinical trials. JAMA Neurol. (2020) 77:1132–40. doi: 10.1001/jamaneurol.2020.1568, 32511687 PMC7281382

[9] Lebrun C Bensa C Debouverie M Wiertlevski S Brassat D de Seze J . Association between clinical conversion to multiple sclerosis in radiologically isolated syndrome and magnetic resonance imaging, cerebrospinal fluid, and visual evoked potential: follow-up of 70 patients. Arch Neurol. (2009) 66:841–6. doi: 10.1001/archneurol.2009.119, 19597085

[10] Polman CH Reingold SC Banwell B Clanet M Cohen JA Filippi M . Diagnostic criteria for multiple sclerosis: 2010 revisions to the McDonald criteria. Ann Neurol. (2011) 69:292–302. doi: 10.1002/ana.22366, 21387374 PMC3084507

[11] Clerici M Saresella M Trabattoni D Speciale L Fossati S Ruzzante S . Single-cell analysis of cytokine production shows different immune profiles in multiple sclerosis patients with active or quiescent disease. J Neuroimmunol. (2001) 121:88–101. doi: 10.1016/S0165-5728(01)00431-3, 11730945

[12] Haki M AL-Biati HA Al-Tameemi ZS Ali IS Al-hussaniy HA. Review of multiple sclerosis: epidemiology, etiology, pathophysiology, and treatment. Medicine (Baltimore). (2024) 103:e37297. doi: 10.1097/MD.0000000000037297, 38394496 PMC10883637

[13] Goyal M Khanna D Rana PS Khaibullin T Martynova E Rizvanov AA . Computational intelligence technique for prediction of multiple sclerosis based on serum cytokines. Front Neurol. (2019) 10:10. doi: 10.3389/fneur.2019.00781, 31379730 PMC6657366

[14] Kallaur AP Oliveira SR Simão ANC Alfieri DF Flauzino T Lopes J . Cytokine profile in patients with progressive multiple sclerosis and its association with disease progression and disability. Mol Neurobiol. (2017) 54:2950–60. doi: 10.1007/s12035-016-9846-x, 27023227

[15] Meyer-Arndt L Kerkering J Kuehl T Infante AG Paul F Rosiewicz KS . Inflammatory cytokines associated with multiple sclerosis directly induce alterations of neuronal Cytoarchitecture in human neurons. J NeuroImmune Pharmacol. (2023) 18:145–59. doi: 10.1007/s11481-023-10059-w, 36862362 PMC10485132

[16] Eshaghi A Young AL Wijeratne PA Prados F Arnold DL Narayanan S . Identifying multiple sclerosis subtypes using unsupervised machine learning and MRI data. Nat Commun. (2021) 12:2078. doi: 10.1038/s41467-021-22265-2, 33824310 PMC8024377

[17] Moazami F Lefevre-Utile A Papaloukas C Soumelis V. Machine learning approaches in study of multiple sclerosis disease through magnetic resonance images. Front Immunol. (2021) 12:700582. doi: 10.3389/fimmu.2021.700582, 34456913 PMC8385534

[18] Rostami A Robatjazi M Dareyni A Ghorbani AR Ganji O Siyami M . Enhancing classification of active and non-active lesions in multiple sclerosis: machine learning models and feature selection techniques. BMC Med Imaging. (2024) 24:345. doi: 10.1186/s12880-024-01528-6, 39707207 PMC11660597

[19] Yousef H Malagurski Tortei B Castiglione F. Predicting multiple sclerosis disease progression and outcomes with machine learning and MRI-based biomarkers: a review. J Neurol. (2024) 271:6543–72. doi: 10.1007/s00415-024-12651-3, 39266777 PMC11447111

[20] Kraus J Kuehne BS Tofighi J Frielinghaus P Stolz E Blaes F . Serum cytokine levels do not correlate with disease activity and severity assessed by brain MRI in multiple sclerosis. Acta Neurol Scand. (2002) 105:300–8. doi: 10.1034/j.1600-0404.2002.1o199.x, 11939943

[21] Fangerau T Schimrigk S Haupts M Kaeder M Ahle G Brune N . Diagnosis of multiple sclerosis: comparison of the poser criteria and the new McDonald criteria. Acta Neurol Scand. (2004) 109:385–9. doi: 10.1111/j.1600-0404.2004.00246.x, 15147460

[22] Witten IH Frank E. Data mining: practical machine learning tools and techniques with Java implementations. SIGMOD Rec. (2002) 31:76–7. doi: 10.1145/507338.507355

[23] Burska A Boissinot M Ponchel F. Cytokines as biomarkers in rheumatoid arthritis. Mediat Inflamm. (2014) 2014:545493. doi: 10.1155/2014/545493, 24733962 PMC3964841

[24] Azizieh F Raghupathy R Shehab D Al-Jarallah K Gupta R. Cytokine profiles in osteoporosis suggest a proresorptive bias. Menopause. (2017) 24:1057–64. doi: 10.1097/GME.0000000000000885, 28609384

[25] Davis JM Knutson KL Strausbauch MA Crowson CS Therneau TM Wettstein PJ . Analysis of complex biomarkers for human immune-mediated disorders based on cytokine responsiveness of peripheral blood cells. J Immunol. (2010) 184:7297–304. doi: 10.4049/jimmunol.0904180, 20495063 PMC2882518

[26] Bellucci G Albanese A Rizzi C Rinaldi V Salvetti M Ristori G. The value of interferon β in multiple sclerosis and novel opportunities for its anti-viral activity: a narrative literature review. Front Immunol. (2023) 14:1161849. doi: 10.3389/fimmu.2023.1161849 37334371 PMC10275407

[27] Bugbee E Wang AA Gommerman JL. Under the influence: environmental factors as modulators of neuroinflammation through the IL-10/IL-10R axis. Front Immunol. (2023) 14:1188750. doi: 10.3389/fimmu.2023.1188750, 37600781 PMC10435745

[28] Iribarren-López A Martins-Almeida L Wells JK Castillo-Triviño T Prada Á Pickett HA . Aging-dependent immunological changes in multiple sclerosis. Front Immunol. (2025) 16:1663526. doi: 10.3389/fimmu.2025.1663526, 41112254 PMC12529361

[29] Dingle K Zimek A Azizieh F Ansari AR. Establishing a many-cytokine signature via multivariate anomaly detection. Sci Rep. (2019) 9:9684. doi: 10.1038/s41598-019-46097-9, 31273258 PMC6609612

[30] Heard BJ Rosvold JM Fritzler MJ El-Gabalawy H Wiley JP Krawetz RJ. A computational method to differentiate normal individuals, osteoarthritis and rheumatoid arthritis patients using serum biomarkers. J R Soc Interface. (2014) 11:20140428. doi: 10.1098/rsif.2014.0428, 24920114 PMC4208376

[31] Boute M Senechal V Gerstenberg A Ramos C. 56 intracellular cytokine profiling and specific immune cell characterization using CyTOF in whole blood for antibody-based cancer immunotherapies. J Immunother Cancer. (2024) 12:56. doi: 10.1136/jitc-2024-SITC2024.0056

[32] Wei F Azuma K Nakahara Y Saito H Matsuo N Tagami T . Machine learning for prediction of immunotherapeutic outcome in non-small-cell lung cancer based on circulating cytokine signatures. J Immunother Cancer. (2023) 11:e006788. doi: 10.1136/jitc-2023-006788, 37433717 PMC10347453

[33] Mouazen B Bendaouia A Abdelwahed EH De Marco G. Machine learning and clinical EEG data for multiple sclerosis: a systematic review. Artif Intell Med. (2025) 166:103116. doi: 10.1016/j.artmed.2025.103116, 40334524

[34] Azizieh F Yilmaz B (2026). Harnessing cytokine signatures and AI for early disease detection and predictive medicine. Intersecting AI and medicine for improved care and administrative efficiency. Kuwait: IGI Global Scientific Publishing. 117–162.

[35] La Rosa F Abdulkadir A Fartaria MJ Rahmanzadeh R Lu P-J Galbusera R . Multiple sclerosis cortical and WM lesion segmentation at 3T MRI: a deep learning method based on FLAIR and MP2RAGE. NeuroImage Clin. (2020) 27:102335. doi: 10.1016/j.nicl.2020.102335, 32663798 PMC7358270

[36] Mahad DH Trapp BD Lassmann H. Pathological mechanisms in progressive multiple sclerosis. Lancet Neurol. (2015) 14:183–93. doi: 10.1016/S1474-4422(14)70256-X, 25772897

